# Evaluating the stability of host-reference gene expression and simultaneously quantifying parasite burden and host immune responses in murine malaria

**DOI:** 10.1038/s41598-023-48066-9

**Published:** 2023-11-29

**Authors:** Daniel J. Browne, Ashton M. Kelly, Jamie Brady, Carla Proietti, Yomani D. Sarathkumara, David J. Pattinson, Denise L. Doolan

**Affiliations:** 1 Centre for Molecular Therapeutics, Australian Institute of Tropical Health and Medicine, James Cook University, Cairns, QLD 4870 Australia; 2 Institute for Molecular Bioscience, The University of Queensland, 306 Carmody Rd, St Lucia, QLD 4072 Australia; 3 Centre for Tropical Bioinformatics and Molecular Biology, Australian Institute of Tropical Health and Medicine, James Cook University, Cairns, QLD 4870 Australia

**Keywords:** Vaccines, Parasite host response, Reverse transcription polymerase chain reaction

## Abstract

The efficacy of pre-erythrocytic stage malaria antigens or vaccine platforms is routinely assessed in murine models challenged with *Plasmodium* sporozoites. Relative liver-stage parasite burden is quantified using reverse transcription quantitative PCR (RTqPCR), which relies on constitutively expressed endogenous control reference genes. However, the stability of host-reference gene expression for RTqPCR analysis following *Plasmodium* challenge and immunization has not been systematically evaluated. Herein, we evaluated the stability of expression of twelve common RTqPCR reference genes in a murine model of *Plasmodium yoelii* sporozoite challenge and DNA-adenovirus IV 'Prime-Target' immunization. Significant changes in expression for six of twelve reference genes were shown by one-way ANOVA, when comparing gene expression levels among challenge, immunized, and naïve mice groups. These changes were attributed to parasite challenge or immunization when comparing group means using post-hoc Bonferroni corrected multiple comparison testing. *Succinate dehydrogenase* (*SDHA*) and *TATA-binding protein* (*TBP*) were identified as stable host-reference genes suitable for relative RTqPCR data normalisation, using the RefFinder package. We defined a robust threshold of 'partial-protection’ with these genes and developed a strategy to simultaneously quantify matched host parasite burden and cytokine responses following immunisation or challenge. This is the first report systematically identifying reliable host reference genes for RTqPCR analysis following *Plasmodium* sporozoite challenge. A robust RTqPCR protocol incorporating reliable reference genes which enables simultaneous analysis of host whole-liver cytokine responses and parasite burden will significantly standardise and enhance results between international malaria vaccine efficacy studies.

Pre-erythrocytic stage malaria vaccine candidates and vaccination platforms are routinely evaluated pre-clinically by quantifying liver-stage *Plasmodium* burden in mouse models^1–3^. Reverse transcription quantitative PCR (RTqPCR) is the gold-standard transcriptome-based diagnostic tool^4^ and is used to quantify liver-stage parasite burden^2,5–12^. RTqPCR analysis of *Plasmodium* liver burden allows the determination of the degree of pre-erythrocytic stage non-sterile protective immunity following sporozoite challenge^6,13,14^. In contrast, quantifying parasitemia following sporozoite challenge with blood-stage diagnostics (i.e., Giemsa-Wright stain microscopy^15^, flow cytometry^16^, or blood-stage RTqPCR^17^) typically represents an 'all-or-nothing' response, and is unable to determine the degree of protection during the liver-stage^2^.

RTqPCR has very high analytical sensitivity and specificity^18,19^. However, inter-study RTqPCR-based results can be inconsistent or irreproducible^20,21^. Variables including sample extraction, RNA isolation and storage, cDNA synthesis and PCR amplification efficiencies may influence RTqPCR measurements^21,22^. These factors can be controlled by reference gene-based relative normalisation. However, a fundamental limitation of relative normalisation is the use of inappropriate or inadequately justified reference genes^18,22,23^, or the selection of a single reference gene^21^. Indeed, robust and reproducible RTqPCR depends upon multiple endogenous reference genes maintaining consistent expression across all experimental conditions^24^. Many conventional reference genes, such as *Glyceraldehyde 3-phosphate dehydrogenase* (*GAPDH*) or *β-actin* (*βACT*), are differentially expressed under certain stimulatory or stressful cellular conditions^22^, including vaccination^25^. Therefore, reference gene validation across all experimental conditions is crucial for reproducible RTqPCR. Despite previous publications describing^2,5,6,26,27^ and using^28–31^ RTqPCR-based liver-stage *Plasmodium* detection strategies, no publication has yet provided a set of stable host reference genes following *Plasmodium* infection or vaccination.

This study describes an integrated dual whole-liver parasite burden and host-cytokine RTqPCR analysis strategy. The ability to use RTqPCR to simultaneously determine important host in vivo immunological responses to challenge and vaccination, as well as quantify matched liver-stage *Plasmodium* burden from an individual animal, is a valuable tool for vaccine development. For example, RTqPCR can measure the transcriptional response to *Plasmodium* challenge of critical immunomodulatory cytokines, such as Interferon-gamma (IFN-γ), Interleukin 2 (IL-2), and Interleukin 10 (IL-10), which are associated with host-protection^32–34^. Unbiased RNA sequencing^35–37^ and RTqPCR protocols^38,39^, have been developed to analyse mRNA responses to *Plasmodium* infection in whole liver, isolated splenocytes^28^ and liver lymphocytes^40^. However, these protocols require additional processing, which preclude matched assessment of parasite burden, did not identify differences in critical protective immunomodulatory cytokines, or have been described following repeated or large sporozoite challenges, which may not be optimal for vaccine antigen testing.

Herein, we report the first assessment of host whole-liver reference gene expression stability for RTqPCR analysis of *Plasmodium* parasite burden. Additionally, we provide an optimised protocol that allows the simultaneous assessment of host-cytokine mRNA responses. Specifically, as a representative immunisation strategy, BALB/c mice were immunised with a DNA prime and intravenous adenovirus 'Prime-Target' strategy^41^, and challenged intravenously with 1,000 *P. yoelii* sporozoites. We developed a robust SYBR® chemistry-based protocol for relative quantification of matched parasite burden and host-cytokine mRNA responses. We identified unstable reference genes with high expression variability between naïve, parasite-challenged, and immunised mice. However, two reference genes *Succinate dehydrogenase (SDHA)* and *TATA-binding protein* (*TBP),* were stable across conditions. Additionally, we found that both challenge and vaccination significantly influenced cytokine expression of several host immunomodulatory cytokines, including *IFN-γ*, *IL-12p40* and *IL-10*. This study provides an optimised protocol that allows simultaneous quantification of host-parasite burden and immune responses to sporozoite challenge and vaccination.

## Materials and methods

### Mouse model and sample generation

#### Immunogens

Full-length *Plasmodium yoelii* circumsporozoite protein (CSP) was synthesised commercially (Genscript, USA) and cloned into a pVR1020 plasmid DNA vector (Vical Inc, USA) downstream from a human cytomegalovirus immediate-early promoter and in-frame with the tissue plasminogen activator signal peptide. Plasmids were purified using an EndoFree plasmid gigaprep kit (Qiagen). A human adenovirus serotype 5 (AdHu5) vector was constructed with a *Py*CSP antigen using pAd/PL-DEST™ Gateway vector system and Gateway LR clonase enzyme (Invitrogen) following the manufacturer's protocol. Linearised plasmids were transfected into Microbix HEK293 cells (Microbix Biosystems Inc., Canada) using a FuGENE HD transfection reagent (Promega, Australia). The virus was then cultured and purified by ultracentrifugation over a caesium chloride gradient, as previously described^42^.

#### Immunisations and parasite challenge

Female BALB/c H-2Dd mice aged 5–7 weeks obtained from the Animal Resource Centre (ARC, Australia) were immunised by intramuscular injection (IM) into the anterior tibialis muscle (50 μl/leg) with 100 μg plasmid DNA (Prime) followed 12 days later with an intravenous injection (IV) into the lateral tail vein (200 μl) of 1 × 10^8^ infectious units (IFU) of respective AdHu5 virus (Target). At 5 weeks post-boost, as tissue resident memory T cells are present^41^, mice were challenged by IV injection of 1,000 cryopreserved *Plasmodium yoelii* 17XNL sporozoites (Sanaria Inc., USA) diluted in 200 µl PBS with 2% naïve mouse serum. Unchallenged and unimmunised (Naïve), sporozoite-challenged infection control (IC), and Prime-Target immunised and challenged (*Py*CSP) mice were studied. All experiments were approved by the Animal Ethics Committee of James Cook University (#A2549), and all procedures were conducted following the 2007 Australian Code of Practice for the Care and Use of Animals for Scientific Purposes, which adheres to the ARRIVE guidelines.

#### Liver harvesting and RNA extraction

All livers were processed identically as previously described^2,6^; however, MagMAX™ *mirVana* Total RNA Isolation Kit (Applied Biosystems) was used for RNA extraction to increase RNA yield^18^. Briefly, whole livers were harvested at 42 h post-challenge in 5 mL MagMAX™ lysis buffer (Applied Biosystems) containing 1% β-2-mercaptoethanol (Sigma-Aldrich, Australia) and homogenised with a TissueRuptor II (Qiagen) homogeniser for 1 min. The lysate was stored at -80 °C. RNA was extracted from 50 µL of liver lysate diluted 1:1 in MagMAX™ lysis buffer, following the manufacturer's recommendations with DNase treatment and elution in 50 μL elution buffer.

#### cDNA synthesis

Extracted mRNA was quantified using a NanoPhotometer® N60 (Implen, München, Germany). RNA (0.4 μg) was then converted to cDNA using the SuperScript™ IV First-Strand Synthesis System (Invitrogen) in 10 μL total volume reactions with random hexamers only with the following modifications from the manufacturer's protocol: cDNA synthesis was conducted with the SuperScript™ reverse transcriptase at half the manufacturers recommended concentration (10U/µL_RNA_), as previously described^43^.

### Quantitative PCR (qPCR)

#### Assay setup

qPCR was performed using ssoAdvanced SYBR® SuperMix (BioRad) following the manufacturer's recommendations (hot start 2 min at 95 °C, followed by 40 cycles of 15 s at 95 °C and 30 s at 60 °C)^18^. Reactions were run at 5 μL total volume amplifying 1 μL sample, as previously described^43^. Reactions were measured by QuantStudio 5 Real-Time PCR Machine running QuantStudio Design and Analysis Software (v1.4.3, Applied Biosystems), using technical triplicates and no template negative controls. Amplification efficiencies were calculated for all qPCR primers by calculating calibration curves from log diluted cDNA of pooled (n = 5) naïve whole mouse liver, or pooled (n = 5) infection control whole mouse liver when testing Py18s primers, as per MIQE guidelines^21^ (Table 1). Cycle threshold values (Ct) were determined with the threshold set in exponential phase amplification at ΔRn0.3. All reactions were followed by a melt curve analysis ensuring primer specificity and contained desalt-grade PrimerBank™^44^ primers (Sigma-Aldrich) run at 500 nM. Reference gene expression stability and whole liver protectivity were evaluated by directly comparing Ct as amplified from 25 ng cDNA per reaction. The host-cytokine response to infection and immunisation was assessed by amplifying 50 ng cDNA per reaction, as per the optimised protocol.

**Table 1:** Primer characteristics. Gene-specific forward and reverse primers acquired from Primer Bank™ database or literature. Assay performance determined as per MIQE guidelines.

Transcript	GenBank accession number	PrimerBank ID/Reference	Forward sequence (5′–3′)	Reverse sequence (5′-3′)	Size (bp)	E′ (%)	R^2^
Reference genes							
*(Mu) 18s*	X00686.1	^57^	GTAACCCGTTGAACCCCATT	CCATCCAATCGGTAGTAGCG	151	110.6	0.99
*GAPDH*	NM_008084	126012538c3	TGGCCTTCCGTGTTCCTAC	GAGTTGCTGTTGAAGTCGCA	178	97.1	0.99
*ACTβ*	X03672.1	^57^	ACTATTGGCAACGAGCGGTT	ACACTTCATGATGGAATTGAATGTAGT	110	97.9	0.99
*RPL13a*	NM_009438	334688867c2	AGCCTACCAGAAAGTTTGCTTAC	GCTTCTTCTTCCGATAGTGCATC	129	100.0	0.99
*PGK1*	NM_008828	6679291a1	ATGTCGCTTTCCAACAAGCTG	GCTCCATTGTCCAAGCAGAAT	164	97.0	0.99
*ALAS1*	NM_020559	23956102a1	TCGCCGATGCCCATTCTTATC	GGCCCCAACTTCCATCATCT	109	101.4	0.99
*SDHA*	NM_025333	31560262a1	GCGGTGGTCACCTTGATCC	CCTCTGTAGAAGCGTCTGAATG	101	99.9	0.99
*IPO8*	NM_001081113	20071797a1	ACGTGACAGTAGATACCAACGC	GCATAGCACTCGGCATCTTCT	115	106.9	0.99
*B2M*	NM_008249	6680223a1	GGCCCATCTTGCATTCTAGGG	CAGGCAACGGCTCTATATTGAAG	100	102.8	0.99
*HPRT1*	NM_013556	7305155a1	TCAGTCAACGGGGGACATAAA	GGGGCTGTACTGCTTAACCAG	142	101.8	0.99
*HMBS*	NM_013551	30794512a1	AAGGGCTTTTCTGAGGCACC	AGTTGCCCATCTTTCATCACTG	78	97.5	0.99
*TBP*	NM_020614	10181156a1	CTTCCTGCCACAATGTCACAG	CCTTTCTCATGCTTGCTTCTCTG	118	99.8	0.99
Cytokine genes							
*IFN-γ*	NM_008337	33468859a1	ATGAACGCTACACACTGCATC	CCATCCTTTTGCCAGTTCCTC	182	99.1	0.96
*TNFα*	NM_013693	7305585a1	CCCTCACACTCAGATCATCTTCT	GCTACGACGTGGGCTACAG	61	107.3	0.98
*IL-2*	NM_008366	1504135a1	TGAGCAGGATGGAGAATTACAGG	GTCCAAGTTCATCTTCTAGGCAC	120	68.8	0.99
*IL-6*	NM_031168	13624311a1	TAGTCCTTCCTACCCCAATTTCC	TTGGTCCTTAGCCACTCCTTC	76	126.3	0.98
*IL-12p40*	NM_008352	6680397a1	TGGTTTGCCATCGTTTTGCTG	ACAGGTGAGGTTCACTGTTTCT	123	86.1	0.96
*IL-1β*	NM_008361	118130747c1	GAAATGCCACCTTTTGACAGTG	TGGATGCTCTCATCAGGACAG	116	101.7	0.99
*IL-10*	NM_010548	6754318a1	GCTCTTACTGACTGGCATGAG	CGCAGCTCTAGGAGCATGTG	105	81.8	0.99
*IL-13*	NM_008355	6680403a1	CCTGGCTCTTGCTTGCCTT	GGTCTTGTGTGATGTTGCTCA	116	65.6	0.94
*TGF-β*	NM_011577	6755774c1	CCACCTGCAAGACCATCGAC	CTGGCGAGCCTTAGTTTGGAC	91	89.6	0.96
17XNL plasmodium yoelii gene							
*Py18s*	XR_004618869.1	^58^	GGGGATTGGTTTTGACGTTTTTGCG	AAGCATTAAATAAAGCGAATACATCCTTAT	104	103.2	0.98

E' Reaction efficiency, R^2^ standard coefficient of determination, (Mu) murine

#### Quantification of host-cytokine expression and parasite burden

Host-cytokine expression was calculated with the standard delta-delta cycle threshold (2^−ΔΔCt^) method relative to the geometric mean of the endogenous control reference genes *SDHA* and *TBP*, as previously described^24^, using naïve mice as the control group. Parasite burden was analysed using a modified 'Fold-reduction' approach (Supp Protocol. 1), wherein several adaptations were made to the standard 2^-ΔΔCt^ protocol. Briefly, since the parasite burden of test groups would be expected to be equal or less than IC mice, the fold-change (i.e., 2^-ΔΔCt^) calculation was inverted to fold-reduction (i.e., 2^ΔΔCt^) using IC mice set as the control group. Since a standard deviation (σ) of *P. yoelii 18s* expression within the IC group was equal to 1 Ct (i.e., 1σ = 0.957 Ct), the threshold of 'partial protection' was set 2σ (i.e., 2 Ct) from the mean. Since the inclusion of qPCR data with Ct > 35 may increase false positive pathogen detection^24,45^, the limit of detection (LOD) of the qPCR assay was set to Ct = 35. Therefore, 'partial protection' is between 2 σ from the mean of the infection control, to the LOD of the assay. 'Sterile protection' is defined as a greater than the LOD of the assay. Both the 'partial protection' and LOD were normalised relative to the experimental geometric mean of endogenous control reference genes *SDHA* and *TBP*.

### Flow-cytometric assessment of parasitemia

Parasitemia was assessed using the flow cytometric assessment of blood (FCAB) assay^16^ from day five post-challenge until the infection had resolved. Briefly, blood from the tail vein was stained with anti-CD71-PE (BioLegend, USA), fixed with PBS containing 4% w/v paraformaldehyde and 0.0067% w/v saponin and then resuspended in buffer containing 0.5 µg/ml bisbenzimide Hoechst 33,342 (Sigma-Aldrich, USA). Flow cytometric analysis was performed on an LSR Fortessa (BD Biosciences, NSW, Australia) using a high-throughput sampler. Post-acquisition data analysis was performed with FlowJo software version 9.4 (Treestar Inc., Ashland, OR, USA). Below 2% Red blood cell (RBC) parasitemia was considered background autofluorescence.

### Statistical analysis

Reference gene expression stability of Ct values was analysed using an Ordinary One-way ANOVA with a Bonferroni-corrected multiple comparisons test against naïve mice. All data were tested for Gaussian distributions with a Shapiro–Wilk normality test. Reference gene expression stability was analysed with RefFinder software as previously described^22^. Briefly, three packages (BestKeeper, geNorm and NormFinder) employed individual statistical approaches to assess reference gene expression stability, which was ranked and tabulated by RefFinder. Host-cytokine expression (Fold-change; 2^-ΔΔCt^) were analysed using a Kruskal–Wallis ANOVA with a Dunn's multiple comparisons test. Analysis was conducted using GraphPad Prism version 7.0 (GraphPad). In all statistical analyses, a *P* < 0.05 was considered significant.

## Results

### The expression of host reference genes is impacted by Prime-target immunisation and *Plasmodium* sporozoite challenge

**Figure 1:**
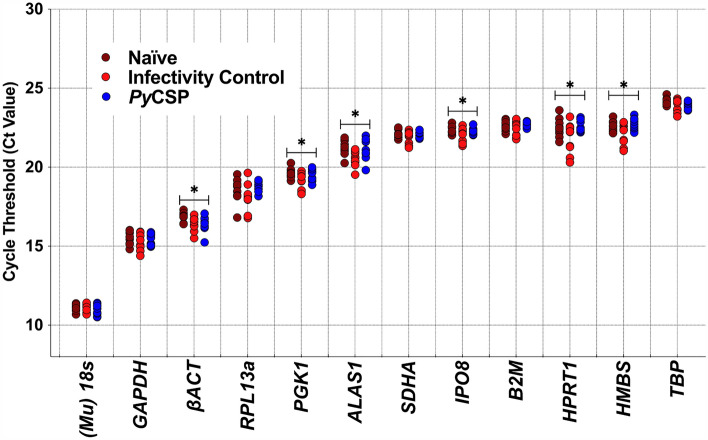
Reference gene expression following 'Prime-Target' Immunisation and *Plasmodium* sporozoite challenge. Groups of BALB/c mice (n = 10/group) included naïve unchallenged (Naïve), naïve with sporozoite challenge (Infection Control), and *Plasmodium yoelii* circumsporozoite protein immunised and sporozoite challenged (*Py*CSP). Where appropriate, mice were intramuscularly immunised with plasmid DNA (Prime), followed 12 days later with an intravenous injection of the respective AdHu5 virus (Target). RNA was extracted from the homogenised whole liver at 5 weeks post-immunisation and 42 h post-challenge with 1,000 *Py*17XNL sporozoites. Cycle threshold (Ct) values were determined from the mean of triplicate replicate qPCR reactions, with the threshold set in exponential phase amplification at ΔRn0.3. Ct values of twelve candidate reference genes from two independent experimental replicates (n = 5/replicate) are shown. Data were analysed using one-way ANOVA with a Bonferroni-corrected multiple comparisons test comparing each group to the naïve mice (* *P* < 0.05).

We determined the stability of expression of twelve commonly reported reference genes^22^ (Table 1) in murine livers following 'Prime and Target' immunisation and *P. yoelii* sporozoite challenge. We found the optimum cDNA concentration to measure reference gene expression was of 25ng or lower, to avoid inhibitory effects seen at higher concentrations (Reaction efficiency (E') > 100%; **Supp **Fig. 1A). A one-way ANOVA identified significant variation in reference gene expression in *βACT (P* = 0.0223), *PGK1* (*P* = 0.0456), *ALAS1* (*P* = 0.0157), *IPO8* (*P* = 0.0284), *HPRT1* (*P* = 0.0449) and *HMBS* (*P* = 0.0334; Fig. 1**)**. Post-hoc analysis found reference gene transcript variation following sporozoite challenge between naïve and IC mice for *βACT (P* = 0.0455), *PGK1* (*P* = 0.0342), *ALAS1* (*P* = 0.0187), *IPO8* (*P* = 0.0168) and *ALAS1* (*P* = 0.0187) genes; and between naïve and *Py*CSP mice for *βACT* (*P* = 0.0211). These data demonstrate that both immunisation and *Plasmodium* challenge impact reference gene expression.

### *SDHA* and *TBP* were identified as the most suitable reference genes for RTqPCR analysis of *Plasmodium* liver-burden and host-cytokine response

To identify the most suitable reference genes for determining both *Plasmodium* liver burden and host-cytokine responses in the liver post-challenge, Ct values for each reference gene (Fig. 1), were ranked for their stability with the RefFinder software package^46^, combining geNorm, NormFinder and BestKeeper analysis (Table 2). *SDHA* and *TBP* were ranked as the most stable genes and had a combined geNorm M stability value of 0.14 which falls below the established threshold of 0.15 for requiring additional reference genes^47^. Therefore, the inclusion of further reference genes beyond the two genes SDHA and TBP was not required. Notably, all packages ranked *SDHA* and *TBP* as the most stable genes, and *RPL13a* as the least stable gene. The most widely used reference gene *GAPDH*^2^ was ranked 6th, 8th, and 9th most stable by geNorm, NormFinder and BestKeeper, respectively.

**Table 2:** Reference gene expression stability as determined by RefFinder. The reference gene stability of 12 potential reference genes was calculated for naïve, sporozoite-challenged, and *Plasmodium yoelii* CSP immunised and sporozoite-challenged BALB/c mice (n = 10/group) by RefFinder software.

	SDHA	TBP	IPO8	PGK1	B2M	HMBS	(Mu) 18s	GAPDH	βACT	HPRT1	ALAS	RPL13a
Rank*	1	1.68	3.46	4.05	4.9	6.12	7.11	7.17	8.91	9.72	10.74	12
GM [C_t_]^#^	21.99	24.01	22.21	19.40	22.57	22.47	10.99	15.34	16.55	22.31	21.01	18.45
AM [C_t_]^#^	22.00	24.01	22.22	19.40	22.57	22.48	11.00	15.35	16.56	22.32	21.02	18.46
Min [C_t_]^#^	21.24	23.21	21.35	18.30	21.78	21.04	9.04	14.39	15.24	20.30	19.53	16.79
Max [C_t_]^#^	22.51	24.61	22.82	20.27	23.04	23.32	11.42	16.02	17.30	23.61	21.99	19.64
SD [± C_t_]^#^	0.23	0.24	0.25	0.34	0.26	0.38	0.31	0.40	0.35	0.50	0.50	0.55
CV [%C_t_]^#^	1.07	0.99	1.12	1.74	1.17	1.71	2.78	2.60	2.11	2.24	2.36	2.95
Min [x-fold]^#^	− 1.69	− 1.73	− 1.82	− 2.14	− 1.73	− 2.71	− 3.85	− 1.93	− 2.47	− 4.02	− 2.79	− 3.16
Max [x-fold]^#^	1.43	1.52	1.52	1.83	1.39	1.80	1.35	1.60	1.68	2.45	1.97	2.29
SD [± x-fold]^#^	1.18	1.18	1.19	1.26	1.20	1.31	1.24	1.32	1.27	1.41	1.41	1.46
S	0.073	0.144	0.177	0.163	0.264	0.222	0.333	0.326	0.451	0.443	0.604	0.631
M	–	0.149	0.181	0.216	0.201	0.272	0.301	0.244	0.344	0.38	0.432	0.478

^#^BestKeeper Statistics, S stability value by NormFinder, M stability value by geNorm GM, geometric mean; AM arithmetic mean; C_t_ cycle threshold; SD standard deviation; CV coefficient of variation. Decreasing Rank, M, and S values signifies increased expression stability; (Mu) murine

### The threshold for partial protection is defined as two standard deviations below the mean of the infection control

**Figure 2:**
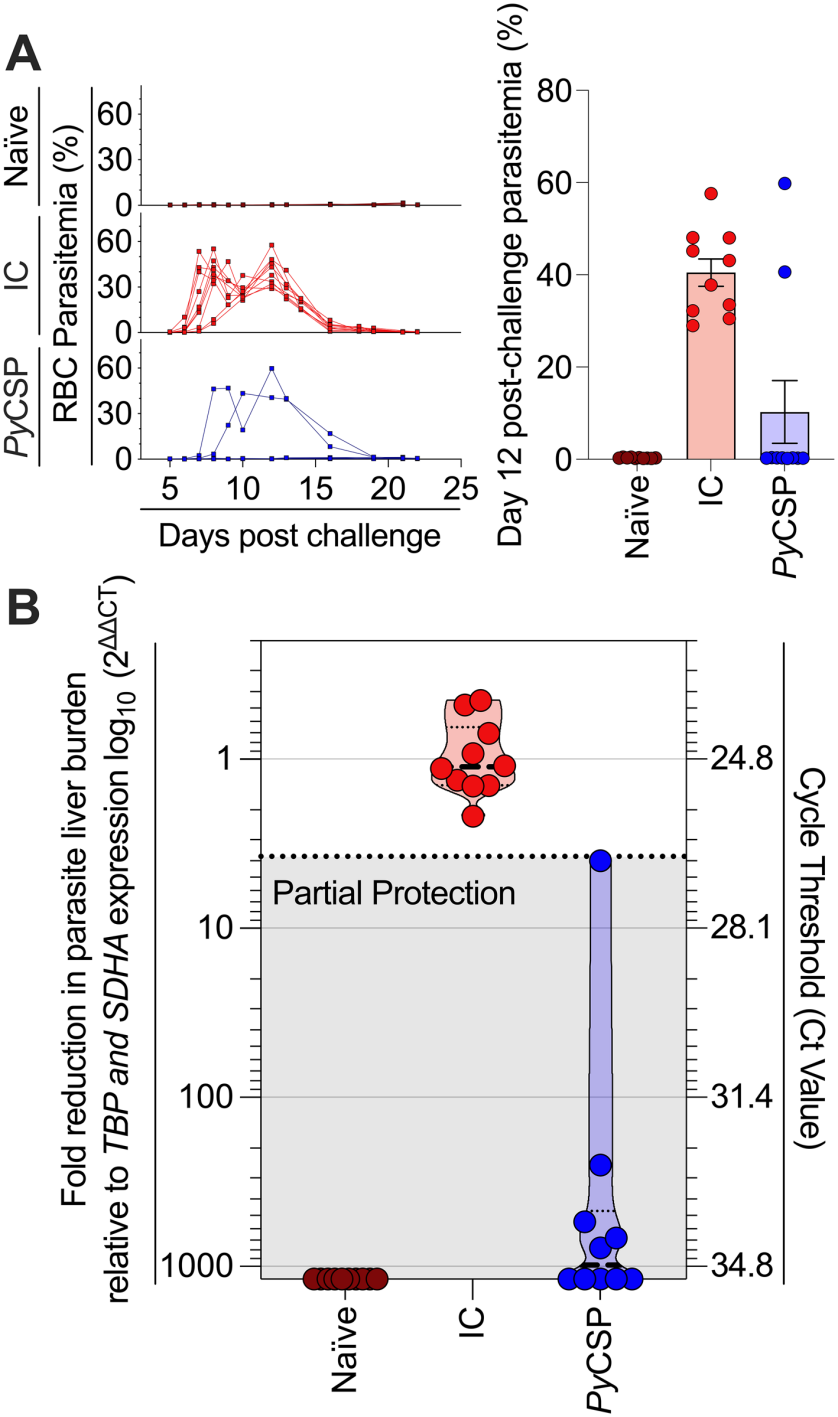
Determination of the threshold of 'partial protection' in the liver stage. BALB/c mice (n = 10/group) were immunised and challenged as described above (Fig. 1 legend): naïve (brown), infection control (IC) sporozoite challenged (red), and *Py*CSP immunised and sporozoite challenged (blue). Parasitemia over the duration of infection and at day 12 post-challenge (**A**) was measured by flow cytometry using the FCAB assay with mean ± technical SEM shown. Liver-stage parasite burden of individual mice was measured at 42 h post-challenge by technical triplicate RTqPCR (**B**). The Ct was determined from the mean of triplicate replicate, with data calculated based on Fold-reduction (2^ΔΔCt^) relative to the Ct geometric mean of the reference genes *TBP* and *SDHA*. Protection was defined as two standard deviations (2 σ = 1.91 Ct) below the mean Ct of the IC (dotted line). The limit of detection (LOD) was *Py18s* Ct = 35 (2^ΔΔCt^ = 1189). Data are pooled from two independent experimental replicates (n = 5/replicate).

When infection was allowed to progress to the blood-stage all IC mice and one in five *Py*CSP mice developed parasitemia (Fig. 2A). Using RTqPCR relative quantification of parasite rRNA in the liver, we could determine both sterile protection (i.e., the absence of *P. yoelii* 18s (*Py18s*) rRNA) and a reduction in parasite burden indicating partial protection (Fig. 2B). A high liver-stage parasite burden was found in all IC mice (*Py18s* Ct mean = 24.78 with σ = 0.96; Fig. 2B). We defined the LOD of the assay as Ct = 35, which provided a fold-reduction dynamic range of the assay (relative to the IC) as 2^ΔΔCT^ = 1189 (Supp Protocol. 1). Furthermore, we defined 2 σ from the *Py18s* Ct mean of the IC as the threshold of partial protection (i.e., Threshold Ct = 26.70 or 2^ΔΔCT^ = 3.77; Fig. 2B), which demonstrated five PyCSP mice were partially protected, and five PyCSP mice were sterilely protected (Fig. 2B). This RTqPCR protocol detected degrees of liver-stage parasite burden, allowing for the interpretation of partial protection.

### Whole-liver host-cytokine expression responds to both immunisation and challenge

**Figure 3:**
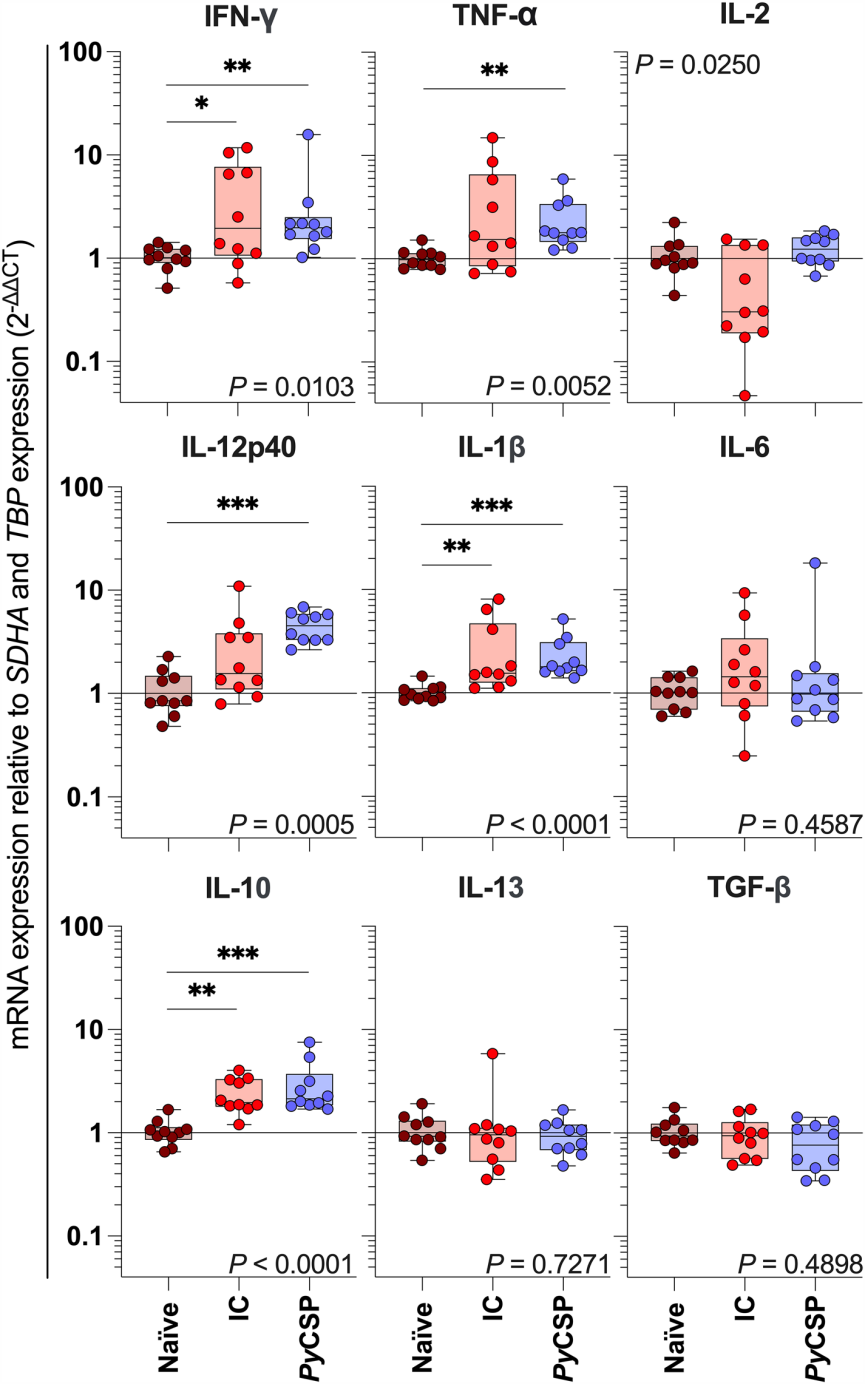
Cytokine expression following Prime-Target immunisation and *Plasmodium yoelii* sporozoite challenge. BALB/c mice (n = 10/group) were immunised with a Prime-Target regimen and challenged with *Py*17XNL sporozoites as previously described (Fig. 1 legend). mRNA expression was assessed by RTqPCR in liver extracts harvested at 42 h post-challenge from naïve, infection control (IC) sporozoite-challenged (red), *Py*CSP-immunised and sporozoite-challenged (blue) BALB/c mice (n = 10/group). Data are pooled from two independent experimental replicates (n = 5/replicate). Fold-change was determined within each experiment with the delta-delta cycle threshold (2^-ΔΔCt^) method relative to the Ct geometric mean of the reference genes *TBP* and *SDHA*. Data were compared with a non-parametric Kruskal–Wallis one-way ANOVA with *P*-value displayed and a post-hoc Dunns-corrected multiple comparisons test comparing test groups to the mean of naïve mice (*, *P* ≤ 0.05; **, *P* ≤ 0.01; ***, *P* ≤ 0.001).

To detect clinically relevant cytokines in the host-whole liver using relative mRNA quantification, we found the optimum concentration of cDNA in the qPCR to detect *IFN-γ* was 50 ng of cDNA per reaction (Supp Fig. 1B). A non-parametric Kruskal–Wallis ANOVA found that the expression of *IFN-γ, TNFα, IL-2, IL-12p40, IL-1β* and *IL-10* was significantly influenced by treatment (*P* = 0.0103, *P* = 0.0052, *P* = 0.0250, *P* = 0.0005, *P* < 0.0001 and *P* < 0.0001 respectively; Fig. 3). Dunn's multiple comparisons testing identified increased expression of *IFN-γ, IL-1β* and *IL-10* in IC mice (*P* = 0.0363, *P* = 0.0027, *P* = 0.0010, respectively; Fig. 3) relative to naïve mice. Likewise, increased expression of *IFN-γ, TNFα, IL-12p40, IL-1β* and *IL-10* was identified in *Py*CSP mice (*P* = 0.0096, *P* = 0.0030, *P* = 0.0002, *P* < 0.0001, and *P* < 0.0001 respectively; Fig. 3) relative to naïve mice. Taken together, these data demonstrate a robust SYBR® chemistry-based RTqPCR protocol for liver-stage *Plasmodium* infection burden testing with matched host-cytokine mRNA response quantification.

## Discussion

Herein, we describe a *Plasmodium yoelii 18s* (*Py18s*) rRNA-specific RTqPCR-based detection strategy with an optimised reference gene selection. This protocol defines 'partial protection' in the liver-stage following a sporozoite challenge and allows matched quantification of host whole-liver cytokine responses. Our assay provides an important update for pre-erythrocytic stage whole-liver *Plasmodium* parasite burden molecular diagnostics.

The inappropriate selection of reference genes is a major contributor to the lack of reproducibility of RTqPCR data^20,21^. Previously published RTqPCR-based relative quantification strategies of *Plasmodium* liver burden are derived from a single reference gene^30^. Indeed, routine or habitual RTqPCR reference gene selection is common across multiple disciplines^48,49^. Using inappropriate reference genes for normalisation may result in the incorrect identification of fully or partially protected animals and misrepresentation of cytokine expression profiles. By analysing the variability of Ct values from 12 commonly cited host reference genes, we identified half were differentially expressed following immunisation and infection, emphasising the importance of systematic reference gene assessment. Although we have established that TBP and SDHA as highly suitable for RTqPCR relative normalisation in our model of *P. yoelii* sporozoite challenge and adenovirus vector-based 'Prime-Target' immunization, it is likely that other stably expressed reference genes may be identifiable with unbiased screening in other models^35–37^. *TBP* and *SDHA* have been identified as stable reference genes for human leukocyte RTqPCR analysis studies^22^.

It is widely acknowledged that reference gene expression stability testing must include all experimental conditions^20,21^, including vaccination^25^ and challenge^50^, as these influence reference gene expression. Our study found statistically significant whole-liver reference gene expression instability in the expression of the commonly cited reference gene *β-actin (βACT*) following a 'Prime & Target' immunisation regimen and parasite challenge. *βACT* can be differentially expressed under inflammatory conditions^51^. therefore, we speculate that the differential expression we observed in whole-liver *βACT* expression may result from antigen-independent adenovirus vector-based inflammation^41^. We found several reference genes were differentially expressed in infection control mice (i.e., *IPO8*, *PGK1* and *ALAS1*) in response to sporozoite challenge. While many key host-parasite liver-stage immunological interactions remain unresolved^52^, innate or innate-adaptive interface immune responses to *P. yoelii* challenge may be driving differential whole-liver cytokine expression. Whilst we have assessed a 'Prime-Target' regimen followed by a 1000 *P. yoelii* sporozoite challenge, other vaccine regimens or challenges involving different sporozoite species or numbers, or other mouse strains will likely require an independent assessment of reference gene expression stability.

A significant advantage of liver-stage parasite burden RTqPCR quantification is the determination of the degree of pre-erythrocytic stage non-sterile protective immunity following sporozoite challenge. To provide a robust method to analyse parasite liver burden data and define 'partial protection' from sporozoite challenge, we made several key adaptations to the standard fold change (2^-ΔΔCt^) method. The first adaptation was to invert the method from fold-change to fold-reduction (2^-ΔΔCt^ *vs* 2^ΔΔCT^) relative to the infection control group. The second adaptation was to define the LOD as Ct = 35. The theoretical LOD (i.e., the lowest amount of measurable analyte) of qPCR is between one and three copies^21^, which under ideal conditions (i.e., a reaction efficiency of 100%) typically reaches cycle threshold (Ct) around cycle 35. Including results > 35 Ct significantly increases the false-positive rate when performing pathogen detection qPCR^24,45^. We set our fold-reduction (i.e., 2^ΔΔCT^) data analysis strategy LOD to Ct = 35 and found a 1189-fold dynamic range from the mean of the IC. The third adaptation was to use this calculated fold dynamic range to provide values to samples from which no amplification occurred. As fold-change RTqPCR analysis is incapable of including 'undefined' samples^24^, an 'u*ndefined*' or 2^ΔΔCT^ > 1189 result was therefore given a value of 2^ΔΔCT^ = 1189. This strategy (Supp. protocol 1) avoids the use of setting non-detect Ct values to a threshold (i.e., Ct = 35), which can introduce substantial bias during normalisation^53^.

The final method adaption was to define a threshold of partial protection. We found the standard deviation (σ) of IC liver parasitemia (*Py18s*) was 0.96 Ct, and the *Py18s* of the IC and the reference genes of all groups were normally distributed. This consistency suggested the I.V. transmissibility of the sporozoites was high. Two standard deviations from the mean typically cover 95% of all intra-group data when normally distributed. Therefore, we defined 'partial protection' as 2 σ from the mean *Py18s* of the IC group to appropriately identify mice with a clinically relevant reduction of parasite liver burden. We did not employ any method to remove technical replicate outliers, as no obvious inappropriate technical variability was observed. However, care must be taken to ensure that results are not biased by high replicate variability, and methods to identify and remove replicate outliers must be reported^21^.

We have optimised a SYBR®-chemistry fold change (ΔΔCt)-based strategy to quantify the whole-liver expression of immunologically important cytokines to facilitate matched host-response and parasite burden assessment. We found the *Py18s* rRNA-specific SYBR® qPCR primers amplified non-specifically in the absence of *Py18s* in the sample. As reported, these readings were excluded based on an incorrect melt curve. It is likely a TAQ-polymerase probe-based assay could eliminate the detection of this non-specific amplification, however, these results demonstrate that careful optimisation is required to ensure the probe does not bind to the non-specific amplicon. We utilised the ΔΔCt method due to its prevalent use in evaluating whole liver parasitemia^2,5,6,26–31^. Although, normalisation methods like the Pfaffl method, which account for primer efficiency, could offer a more rigorous analysis of gene expression data^54^.

The simultaneous quantification of liver parasite burden and host-cytokine response in a standardised protocol is an important addition to pre-erythrocytic stage vaccine development, as this technique will increase the reproducibility of studies investigating the host immune response elicited during the pre-erythrocytic stage to vaccination and challenge^40^. The critical effector molecule of adaptive immunity to sporozoite challenge appears to be Interferon-gamma (IFN-γ) released by CD8^+^ T cells^34,55^, and Th1 CD4^+^ T cells secreting IFN-γ and Interleukin-2 (IL-2)^32,33,56^. The mRNA expression profiles of IFN-γ and many other rapidly produced and secreted cytokines are relatively highly correlated to protein production^43^. Therefore, transcriptomic quantification of host-cytokine responses will inform functional efforts to understand the immunological response following vaccination. A protocol that can evaluate mRNA expression of essential host effector genes following a challenge of 1,000 *P. yoelii* sporozoites is expected to provide the sensitivity required for most *P. yoelii* vaccine challenge models. Furthermore, it is anticipated that a similar strategy to quantify whole organ cytokine response could be applied to other immunisation, mouse strain, *Plasmodium* species, or other pathogen challenge rodent models.

Here, we present a protocol for the robust analysis of primary liver-stage *Plasmodium* infection and pre-erythrocytic stage immunity burden testing. We demonstrate that *P. yoelii* infection and 'Prime-Target' immunisation influence reference gene expression and identify *SDHA* and *TBP* as optimal reference genes for relative RTqPCR normalisation. We have established a criterion for defining partially protective immunity to infection and provide a customised fold-reduction method to provide a LOD and account for 'u*ndefined*' measurements. This assay is suitable for studying whole-liver host-cytokine mRNA responses, which are matched with a parasite-burden readout. This protocol is designed to be broadly adaptable across various murine models. While we anticipate the need for reference gene optimization may vary depending on the specific model, the protocol presented herein offers a systematic framework for identifying stable RTqPCR reference genes in mouse whole liver, determining ‘partial’ and ‘sterile’ protection, and assessing the expression of critical matched host immunomodulatory genes. This report provides an important update for further trials evaluating pre-erythrocytic stage whole-liver *Plasmodium* parasite burden and host response and highlights the importance of thorough selection of reference genes for RTqPCR.

### Supplementary Information

Supplementary Information 1.

Supplementary Information 2.

## Data Availability

All data supporting the findings of this study are available within the paper and its Supplementary Information.
