# Supplementary material for: Evaluating the stability of host-reference gene expression and simultaneously quantifying parasite burden and host immune responses in murine malaria
Source: Sci Rep. 2023 Nov 29;13:21071. doi: 10.1038/s41598-023-48066-9 (PMC10687243; doi:10.1038/s41598-023-48066-9)
Supplement: Supplementary file 2 — Supplementary Information 2. [file 41598_2023_48066_MOESM2_ESM.docx]

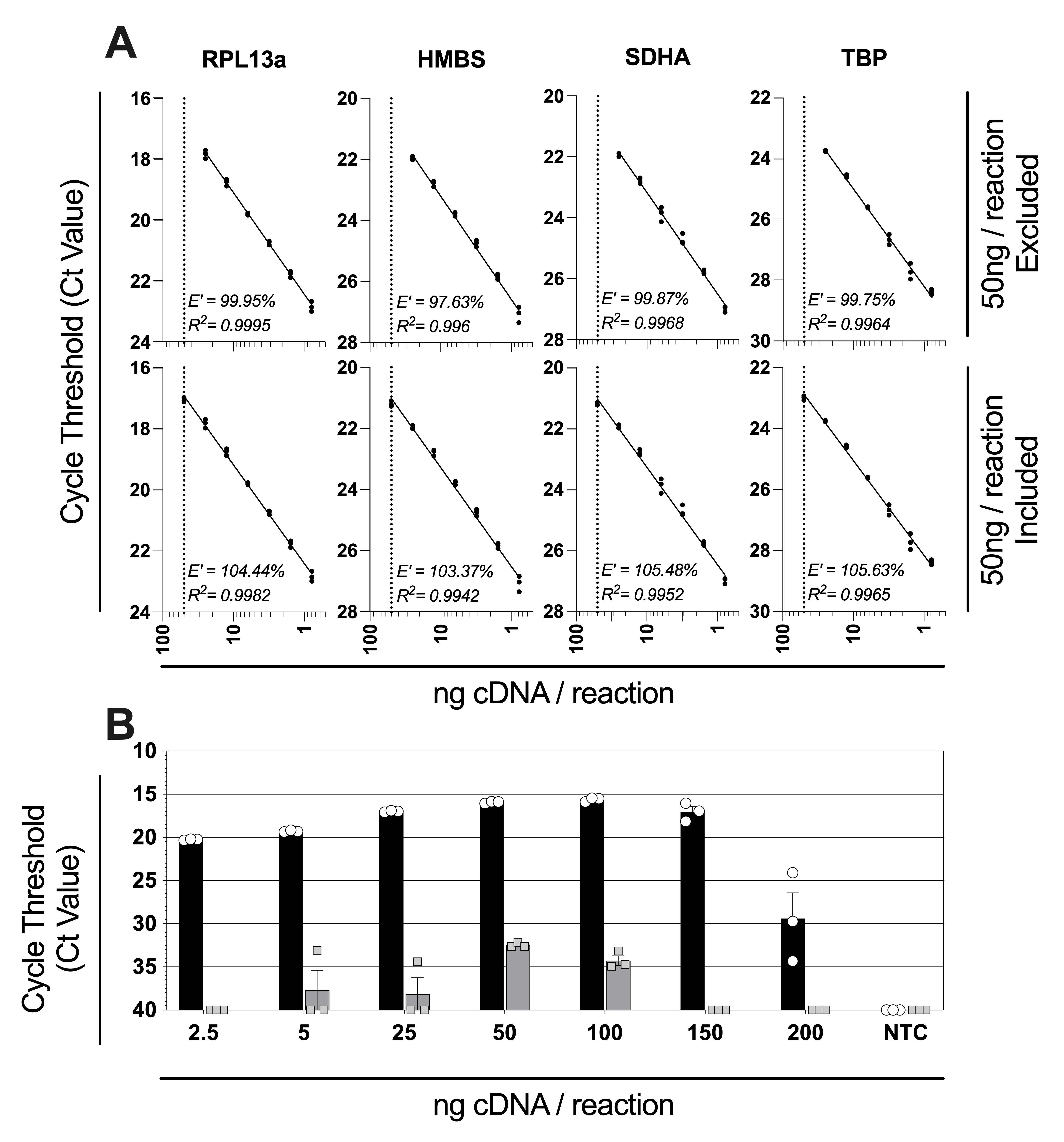


**Supplementary Figure 1. Optimisation of cDNA concentration in reaction for reference gene assessment and cytokine detection.** (**A**) The reaction efficiency (E’) and the coefficient of determination (R^2^) were calculated as per the MIQE guidelines from Log titrations of cDNA testing reference genes RPL13a, HMBS, SDHA and TBP with 50ng included (bottom) and 50ng excluded (top). (**B**) mRNA expression of reference gene RPL13a (black bars, white spots) and cytokine IFN-γ (grey bars, grey squares) from pooled (n=5) naïve whole mouse liver; and no-template negative control (NTC). Shown is the cycle threshold value (Ct Value), with undetermined values receiving a value of 40, as measured by RTqPCR. Individual technical replicates (spots and squares) are shown alongside the technical replicate mean (bars) ± technical SEM.
